# The influence of maternal reflective functioning and parenting behavior on infant development in the context of perinatal intimate partner violence: a study protocol

**DOI:** 10.1186/s40359-023-01191-6

**Published:** 2023-05-19

**Authors:** Inês Jongenelen, Tiago Miguel Pinto, Raquel Costa, Rita Pasion, Ana Morais, Sandra Henriques, Diogo Lamela

**Affiliations:** 1 HEI-Lab: Digital Human‐Environment Interaction Labs, Lusófona University, Porto, Portugal; 2 EPIUnit, Instituto de Saúde Pública, Universidade do Porto, Porto, Portugal; 3 Laboratório para a Investigação Integrativa e Translacional em Saúde Populacional (ITR), Porto, Portugal

**Keywords:** Intimate partner violence, Parental reflective functioning, Parenting behavior, Infant development

## Abstract

**Background:**

Intimate partner violence (IPV) affects 25% of children under the age of five worldwide, yet the impact of perinatal IPV and its underlying mechanisms on infant development remains poorly understood. IPV indirectly affects infant development through the mother’s parenting behavior, but research on maternal neuro and cognitive processes, such as parental reflective functioning (PRF), is scarce, despite its potential as an unfolding mechanism. The objective of our study, Peri_IPV, is to examine the direct and indirect pathways linking perinatal IPV and infant development. We will analyze the direct impact of perinatal IPV on mothers’ neuro and cognitive parental reflective functioning (PRF) and parenting behavior during the postpartum period, the direct impact of perinatal IPV on infant development, and whether maternal PRF mediates the link between perinatal IPV and parenting behavior. We will also explore the mediation role of parenting behavior in the association between perinatal IPV and infant development and whether the impact of perinatal IPV on infant development occurs through the links between maternal PRF and parenting behavior. Finally, we will examine the moderation role of mother’s adult attachment in the impact of perinatal IPV on maternal neuro and cognitive PRF and parenting behavior during the postpartum period, as well as on infant development.

**Methods:**

Our study will use a multi-method, prospective design to capture different levels of PRF, parenting behavior, and infant development. Three-hundred and forty pregnant women will participate in a 4-wave longitudinal study from the 3rd trimester of pregnancy to 12 months postpartum. In the 3rd trimester and 2 months postpartum, women will report on their sociodemographic and obstetric characteristics. In all assessment waves, mothers will complete self-reported measures of IPV, cognitive PRF, and adult attachment. At 2 months postpartum, women’s neuro PRF will be monitored, and at 5 months postpartum, their parenting behavior will be assessed. The infant-mother attachment will be assessed at 12 months postpartum.

**Discussion:**

Our study’s innovative focus on maternal neuro and cognitive processes and their impact on infant development will inform evidence-based early intervention and clinical practices for vulnerable infants exposed to IPV.

## Background

IPV is a critical public health issue that affects around 25% of women globally during the perinatal period [9]. UNICEF estimates that one in four children under the age of five (176 million in total) live with a mother who has been exposed to IPV [1]. Infants born to women exposed to IPV during pregnancy have an increased risk for negative neonatal outcomes [2], including poorer offspring regulatory outcomes [3]. Additionally, these women are less likely to seek healthcare services, despite experiencing more physical and mental health problems [3].

Meta-analyses consistently conclude that children’s exposure to IPV is associated with a higher risk of several negative developmental outcomes, including internalizing and externalizing symptomatology, perceptual and cognitive problems, academic and interpersonal difficulties, and insecure or disorganized attachment to their caregivers [4, 5]. Nonetheless, studies in this research field have mostly been conducted with children in early and middle childhood and adolescence [6]. As a result, little is known about the adverse consequences of exposure to IPV during infancy to developmental tasks and mechanisms underlying the emergence of negative developmental and mental health outcomes.

While research on perinatal IPV remains limited, seminal evidence indicates that it has a detrimental impact on infants’ physical and socioemotional development, particularly by age 1 [3]. Infants exposed to IPV are at higher risk of mental health problems such as poor sleep regulation, increased irritability, trauma symptoms, hyperarousal, and fear [7]. Furthermore, perinatal IPV interferes with the development of secure attachment between the infant and mother [8]. IPV during pregnancy is a risk factor for insecure attachment at age 1, which predicts the stability of insecure attachment at age 4 [8]. However, these findings do not provide a full understanding of the underlying mechanisms through which IPV interferes with infants’ development during the first year of life.

A major limitation of previous research concerns the fact that it fails to account for the sensitive period of the first year, during which emotional and behavioral self-regulatory functions develop and has a significant impact on later developmental and mental health outcomes [3, 9]. In order to explain the mediating process by which IPV affects infant development, some researchers using the developmental psychopathology framework suggest that IPV impairs mothers’ abilities to provide sensitive, responsive, and reliable care to their infants [10]. However, research examining the mediation models that link IPV, parenting, and children’s outcomes has produced complex and counterintuitive results [11, 12].

Some studies have found that exposure to IPV is a significant risk factor for less sensitive maternal behavior during infancy, with IPV being strongly associated with harsh and intrusive parenting, dyssynchronous parent-infant interactions, and subsequent socio-emotional difficulties in infants [13, 14]. In contrast, others have found that positive parenting practices in mothers exposed to IPV, such as warmth and support, predicted fewer mental health problems in their children [11].

More recently, different parenting patterns have been identified in a sample of mothers exposed to similar levels of IPV [15]: (1) a spillover pattern, in which mothers transferred IPV-related negativity and emotional distress to the mother-child relationship, and (2) a compartmentalized pattern, in which mothers demonstrated effective parenting practices. These findings provide a compelling integrative approach to reconcile previous contradictory results by revealing different profiles in a sample with similar levels of IPV exposure.

In this context, it is important to note that mothers’ mental health problems may serve as a potential explanatory mechanism for how IPV can differentially impact parenting behavior and subsequent children’s developmental outcomes [13, 16]. While previous empirical work has made significant strides in understanding the interplay between IPV and parenting, less is known about the potential role of neuro and cognitive parenting-related dimensions in explaining the variability in maternal parenting behavior and infant development.

Recent studies have hypothesized that PRF is a maternal cognitive process that links women’s exposure to IPV and parenting behavior [17]. PRF involves a caregiver’s ability to understand and reflect on their own internal mental experiences, their child’s internal mental experiences, and how both relate to contextualize the child’s behavior [18]. This mentalizing capacity, which is an overt manifestation of an individual’s ability to conceptualize, is closely related to parental attachment [19, 20] and is a strong predictor of sensitive and responsive parenting [20] as well as infant development, including secure attachment, emotional regulation ability, and lower aggressive behavior [21–23]. Some studies on mother-toddler dyads have suggested that exposure to IPV can negatively affect mothers’ PRF and, ultimately, maternal sensitivity [24].

Despite the importance of PRF, only a few studies have analyzed the neuro mechanisms underlying this process and its influence on maternal parenting behavior and infant development. These studies have identified brain areas such as dorsolateral prefrontal and temporal regions that subserve PRF faculties for mother-infant bond formation, highlighting the importance of assessing both neural and cognitive PRF [17, 25]. It has also been suggested that exposure to IPV may alter both neuro and cognitive PRF [17].

Our study aims to move forward in state-of-the-art by examining the unexplored interplay between perinatal IPV and maternal neuro and cognitive PRF in predicting maternal parenting behavior and infant development. The PERI_IPV study provides multimodal and longitudinal data on the conjoint operation of neuro, cognitive, and behavioral dimensions of maternal parenting under IPV exposure impact on infant development during the first year of life.

**Fig. 1:**
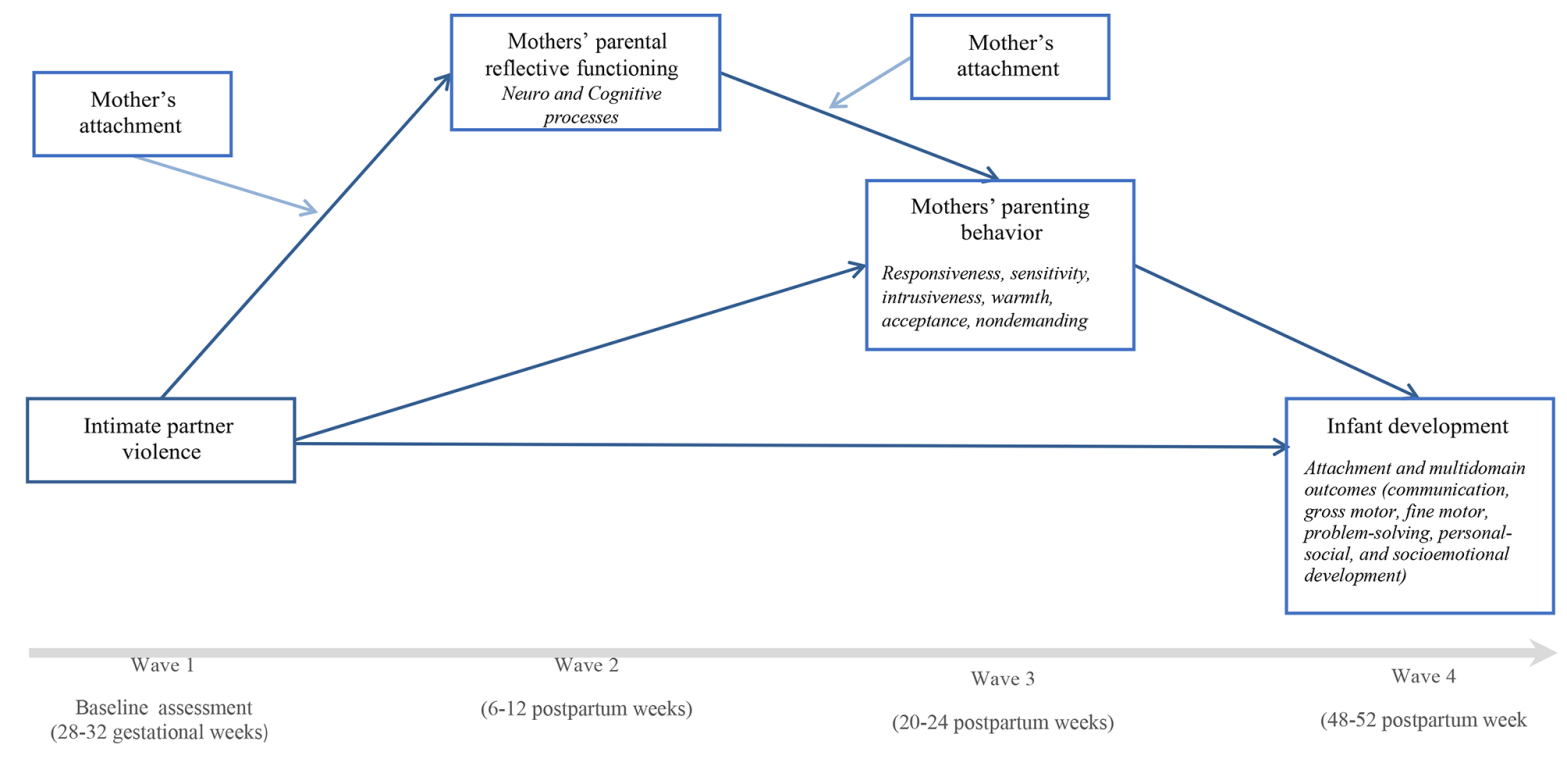
Study model. Model to test the project objectives: **(1)** the direct impact of perinatal IPV on mothers? neuro and cognitive PRF, and on parenting behavior; **(2)** the direct impact of perinatal IPV on infant development at 1 year of age; **(3)** whether mothers? neuro and cognitive PRF mediate the link between perinatal IPV and mother?s parenting behavior; **(4)** the mediator role of mother?s parenting behavior in the association between perinatal IPV on infant development; **(5)** if the impact of perinatal IPV on infant development occurs through the links between mothers? neuro and cognitive PRF, and parenting behavior, and **(6)** the moderator of mother?s adult attachment in the impact of perinatal IPV on mother?s neuro and cognitive PRF and parenting behavior during the postpartum period, and on infant development

The main aim of the Peri_IPV is to analyze the impact of perinatal IPV on infant development. Specific aims include examining: (1) the direct impact of perinatal IPV on mothers’ neuro and cognitive PRF and parenting behavior; (2) the direct impact of perinatal IPV on infant development at one year of age; (3) whether mothers’ neuro and cognitive PRF mediate the link between perinatal IPV and mother’s parenting behavior; (4) the mediator role of mother’s parenting behavior in the association between perinatal IPV on infant development; (5) if the impact of perinatal IPV on infant development occurs through the links between mothers’ neuro and cognitive PRF, and parenting behavior, and (6) the moderator of mother’s adult attachment in the impact of perinatal IPV on mother’s neuro and cognitive PRF and parenting behavior during the postpartum period, and on infant development (Fig. 1).

## Methods/Design

### Participants

We expect to contact at least 459 mothers. Accounting for possible refusals and dropouts, we expect to enroll in the study at least 340 mother-infant dyads with all the assessment waves completed. Mothers will be eligible if they are 18 years of age or older, with a single gestation. Infants born with ≤ 37 gestational weeks, birth weight ≤ 2500 g, or congenital abnormalities will be excluded. A priori power calculations revealed that the sample size is adequate to conduct the structural equation modelling (effect size = 0.30, power = 0.95, number of latent variables = 1, number of observed variables = 5, p < 0.05, *N* = 100) to address the proposed aims [26].

### Procedures

The study obtained ethical approvals from the involved institutions (NV 337; CES 279_2022). Pregnant women will be systematically recruited at the maternal and newborn care facilities during the 3rd trimester of pregnancy. A researcher will approach eligible women at the Outpatient Obstetrics Unit and explain the study’s objectives and procedures, inviting them to participate. This study has a longitudinal design with four assessment waves: (1) 3rd trimester of pregnancy (28–32 gestational weeks), (2) two months postpartum (6–12 postpartum weeks), (3) five months postpartum (20–24 postpartum weeks), and (4) 12 months postpartum (48–52 postpartum weeks).

During the third trimester and at two months postpartum, participants will report on their sociodemographic and obstetric characteristics, as well as infant biometric data. At all assessment waves, participants will complete self-reported measures to assess intimate partner violence (IPV), cognitive PRF, adult attachment, and mental health problems (including anxiety, depression, and IPV-related PTSD symptoms). Participants will also report on their infant’s development in all postpartum assessment waves.

Laboratory procedures will be conducted with the women and their infants at the University labs at two, five, and 12 months postpartum. Specifically, women’s neuro PRF will be monitored and recorded at two months postpartum during the Still-Face Paradigm [27](SFP). Mother’s and infant’s brain functional activation patterns will be recorded simultaneously using a fNIR hyper-scanning acquisition setup. Data will be collected on a two-wavelength (760 and 850 nm) continuous-wave Photon Cap C20 system, Cortivision, Poland (sampling rate: 92 Hz). The 32 channels are composed of 16 LEDs sources and 10 SiPD detectors. Galvanic skin response will also be measured as a metric of emotional activation/distress in SFP using a wireless device (James II, Mindprober, PT, sampling rate: 500 Hz). Both apparatuses are non-invasive, well-validated, and widely used in naturalistic settings [28, 29]. They require minimal setup time (i.e., both are wireless) and capture high-quality biometric signals (i.e., artifact-resistant and high signal-to-noise ratio). Conditions of the SFP paradigm (play episode still episode and reunion episode) will be triggered to both devices.

We will observe and videotape women’s parenting behavior at five months postpartum in a 5-minute face-to-face interaction. The Global Rating Scales of Mother-infant Interaction (GRS) will be used to code the interaction [30, 31]. At 12 months postpartum, infant-mother attachment will be observed and videotaped during the Strange Situation Procedure [32] (SSP). Two trained researchers will independently score the parenting behavior and infant-mother attachment, and agreement will be assessed. The researchers will be blinded to other information about the participants. Table 1 presents the study’s design, assessment waves, variables, and measures.

**Table 1:** Study design, assessments waves, variables, and measures

Study variables	Pregnancy	Postpartum		
	3rd. trimester (28–32 gestational weeks)	2 months (6–12 weeks)	5 months (20–24 weeks)	12 months (48–52 weeks)
Mother	Sociodemographic and obstetric data^1^	Sociodemographic and obstetric data^1^		
	IPV-related PTSD symptoms^2^ Anxiety symptoms^3^ Depressive symptoms^4^	IPV-related PTSD symptoms^2^ Anxiety symptoms^3^ Depressive symptoms^4^	IPV-related PTSD symptoms^2^ Anxiety symptoms^3^ Depressive symptoms^4^	IPV-related PTSD symptoms^2^ Anxiety symptoms^3^ Depressive symptoms^4^
	Perinatal IPV^5^	Perinatal IPV^5^	Perinatal IPV^5^	Perinatal IPV^5^
	Mother’s attachment^6^	Mother’s attachment^6^	Mother’s attachment^6^	Mother’s attachment^6^
Mother-Infant		Neuro PRF^7^ Cognitive PRF^8^	Cognitive PRF^8^	Cognitive PRF^8^ Infant-mother attachment^9^
			Parenting Behavior^10^	
Infant		Infant development^11^	Infant development^11^	Infant development^11^

**Notes.** 1.Sociodemographic and obstetric data; 2. PTSD-Checklist for the DSM-5 (PCL-5); 3. State-Trait Anxiety Inventory (STAI-S/T); 4. Edinburgh Postnatal Depression Scale (EPDS); 5. Violence Against Women Instrument (VAWI); 6. Experiences in Close Relationships–Relationship Structures questionnaire (ECR-RS); 7. Still Face Paradigm (SFP); 8. Prenatal and postpartum Parental Reflective Functioning Questionnaire (Pre-PRFQ and Pos-PRFQ); 9. The Strange Situation Procedure (SSP); 10. Global Rating Scales of Mother-infant Interaction (GRS); 11. The Ages & Stages Questionnaires (ASQ-3); Ages & Stages Questionnaires: Social-Emotional (ASQ:SE).

### Measures

#### Sociodemographic and Obstetric Information

Sociodemographic and obstetric data (age, marital status, professional status, country of birth, country of residence, history of mental health problems and treatment, parity, gestational age at birth, type of birth, maternal/neonatal complications) will be collected using a self-reported questionnaire.

#### Perinatal IPV

The Violence Against Women Instrument (VAWI) will be used by [33], which assesses controlling behaviors, psychological violence, physical violence, and sexual violence.

#### Parental reflective functioning

##### **Neuro**

Data will be collected during the Still Face Paradigm [27], a procedure that allows assessing mother’s sensitivity in response to infant signals both in non-distress (during play episode) and distress (during reunion episode) situations. It comprises three episodes: (1) face-to-face play episode (5 min), (2) still-face episode (2 min), and (3) reunion episode (2 min). Galvanic Skin Conductance (neurophysiological data) is collected using MindProber’s biometric sensors. Cortivision fNIR solutions provide a hyperscanning mode to measure between-person coordination brain activity dynamics (neuroimaging), as indicated by the alignment of two independent signals across time [29].

##### Cognitive

The prenatal and postpartum Parental Reflective Functioning Questionnaire [20, 34] (Pre-PRFQ and Pos-PRFQ) will be used. Pre-PRFQ has three subscales: opacity of mental states, reflecting on the fetus-child, and the dynamic nature of mental states; Pos-PRFQ comprises three subscales: pre-mentalizing modes, certainty about mental states, and interest and curiosity.

### Parenting behavior

A 5-minute face-to-face play episode will be recorded, and parenting behaviors will be assessed using the Global Rating Scales of Mother-infant Interaction [30, 31](GRS), which assesses the quality of maternal behavior, infant behavior, and overall interaction.

### Adult attachment

The Experiences in Close Relationships–Relationship Structures questionnaire [35] (ECR-RS) will be used to assess avoidance and anxiety orientations of adult attachment.

### Infant development

The Ages & Stages Questionnaire [36](ASQ-3) and the Ages & Stages Questionnaire: Social-Emotional [34](ASQ:SE) will be used. The ASQ-3 includes five domains: communication, gross motor, fine motor, problem-solving, and personal–social and the ASQ:SE focuses exclusively on infant social-emotional development.

### Infant-mother attachment

The Strange Situation Procedure [32](SSP) will be conducted and videotaped. Two experts will analyze the videos and classify infants as secure, insecure-avoidant, insecure-resistant, and disorganised [32, 37].

### Mental health problems

The State-Trait Anxiety Inventory [38] (STAI-S/T) will be used to assess anxiety symptoms, the Edinburgh Postnatal Depression Scale [39] (EPDS) will be used to assess depressive symptoms, and the Post-Traumatic Stress Disorder (PTSD) -Checklist for the DSM‐5 (PCL-5) [40] will be used to assess IPV-related PTSD symptoms.

### Data analysis

The aims of Peri_IPV will be achieved by using descriptive and multivariate analysis and longitudinal path analysis (LPA) using structural equation modelling. Specifically, unadjusted and adjusted multilevel linear regression models will be performed to analyze specific aims 1 and 2. A LPA model will be tested to analyze specific aims 3 to 6, according to the proposed study model (Fig. 1). The model will be constructed using observed variables, and maximum likelihood estimation will be applied. The model will be submitted to Wald test modification indices to increase goodness of fit indices [41]. Indices from different classes will be used to assess model goodness of fit. Mediation links will be identified through the decomposition of effects, and mediation and moderation links will be tested following guidelines [42].

## Discussion

The Peri_IPV study aims to investigate the impact of perinatal IPV on infant development at one year of age and the role of neuro, cognitive, and behavioral dimensions of parenting in this association. The study’s findings will inform the development of evidence-based early intervention programs and clinical practice for vulnerable infants who have been directly or indirectly exposed to IPV. The results of the Peri_IPV study are crucial for expanding the scientific knowledge that policymakers rely on to develop, evaluate, and implement effective policies for preventing the short- and long-term effects of IPV exposure on infant developmental pathways. The study’s formal partnership with the Portuguese Association for Victim Support will help disseminate the results among political stakeholders, professionals, and the community. Altogether, Peri_IPV presents a multiplier effect on combating social and health inequalities in this highly vulnerable population.

The study’s aims are informed by developmental science (e.g., attachment theory) and theoretical models of the developmental psychopathology frameworks. By examining the interplay between perinatal adverse conditions, cognitive and behavioral dimensions of parenting, and infant attachment and self-regulatory functioning, this study will also advance knowledge in developmental psychology. Moreover, the findings will contribute to evidence-based clinical practice in early intervention for mothers and infants exposed to IPV. Additionally, the multidomain assessment protocol used in this study can be a valuable tool in forensic psychological assessment of infants exposed to IPV. By examining the association between neuro PRF, parenting behavior, and infant development, the results of this study will also advance the field of basic psychology [43].

Peri_IPV findings provide evidence of the impact of perinatal IPV on infant development, informing psychological interventions targeting IPV families focusing on the perinatal period. Violence against women and children is considered one of the most important social and economic barriers to sustainable development across the globe, conditioning healthy lives, gender equality, and peaceful and inclusive societies. The United Nations 2030 Agenda for Sustainable Development Goals (SDGs) highlights the prevention of all forms of violence against women and children as a central pillar to achieve all these goals. Results contribute to designing more effective violence risk and mental health assessments of women and designing more timely and effective interventions attending to families’ needs. This aligns with goal 3.4 - reduce by one-third premature mortality from non-communicable diseases through prevention and treatment and promote mental health and wellbeing. Results provide evidence on mothers’ perinatal mental health disorders associated with prenatal IPV, which contribute to developing more effective screenings to identify mothers at risk and who may need specialised perinatal mental health and social care, also aligned with goal 3.4. Peri_IPV also aligns with SDG 5, regarding gender equality and the empowerment of women and girls, specifically goal 5.2 - eliminate all forms of violence against all women and girls in public and private spheres. In addition, Peri_IPV aligns with SDG 16, which fosters peace, justice, and strong institutions for sustainable development claims in goal 16.2 to eliminate abuse, exploitation, trafficking, and all forms of violence against children. High macro and individual costs of IPV on women and their children have been internationally reported by the World Health Organization, with billions of dollars being spent each year within the health and judicial system. Portuguese authorities actively incorporate these SDGs in the legislation and health and social systems policies. As such, Peri_IPV outcomes contribute to social and healthcare providers’ strengthened capacity to respond effectively to the needs of women and children affected by violence. Peri_IPV will provide evidence-based guidelines and implications for prevention and intervention in order to minimize the personal and social costs of exposure to IPV, strengthening and scaling up the contribution of psychological science towards the accomplishment of SDGs 3.4, 5.2, and 16.2 in Portugal, with implications for international scientific and professional communities.

The Peri_IPV results will be communicated and disseminated within the non-scientific and scientific communities. Within the non-scientific community, periodical newsletters are developed and posted on relevant social networks, namely key findings, fact sheets, or research briefs. Within the scientific community, the Peri_IPV results will be communicated and disseminated in national and international research events, international papers, and the presentation of oral and poster communications at national and international conferences and through master and Ph.D. thesis.

## Data Availability

Available upon request to the corresponding author.

## List of abbreviations

ASQ:SE: Ages & Stages Questionnaires: Social-Emotiona
SQ-3: Ages & Stages Questionnaire
DSM-5: Diagnostic and Statistical Manual of Mental Disorder
ECR-RS: Experiences in Close Relationships–Relationship Structures questionnair
EPDS: Edinburgh Postnatal Depression Scale
fNIR: Functional Near-infrared Spectroscopy
GRS: Global Rating Scales of Mother-infant Interaction
IPV: Intimate Partner Violence
LPA: longitudinal path analysis
LU: Lusófona University
PRF: Parental Reflective Functioning
PCL-5: PTSD-Checklist for the DSM‐5;Disorder
PRFQ: Parental Reflective Functioning Questionnaire
PTSD: Post Traumatic Stress
SFP: Still-Face Paradigm
SSP: Strange Situation Procedure
SDG: sustainable development goals
VAWI: Violence Against Women Instrument
